# Occupational stress of elementary school teachers after eased COVID-19 restrictions: a qualitative study from China

**DOI:** 10.3389/fpsyg.2023.1183100

**Published:** 2023-05-25

**Authors:** Yujing Yao, Jie Xu

**Affiliations:** Jing Hengyi School of Education, Hangzhou Normal University, Hangzhou, China

**Keywords:** job stress, job burnout, primary school teacher, COVID-19, qualitative study

## Abstract

**Background:**

On December 7, 2022, China optimized its virus response and significantly shifted its epidemic policy by downgrading COVID management and gradually restoring offline teaching in schools. This shift has brought many impacts on teachers.

**Aims:**

Through qualitative research of thematic analysis, this paper studies the occupational pressure of primary school teachers in China after the shift in epidemic policy.

**Methods:**

Two recruitment methods are adopted for this study. One was to email the principals of several primary schools in Zhejiang Province to introduce the research project and indicate the idea of recruiting participants. With their help, we have found teachers who volunteer to participate. The second was to release recruitment information in the network forum (e.g., online teacher forums) to find volunteer participants. Through semi-structured interviews and diaries, 18 primary school teachers from different regions and schools in Zhejiang Province were interviewed. All responses in the interviews were transcribed anonymously. Braun and Clarke’s thematic analysis was used to analyze the participants’ responses.

**Results:**

Eighteen participants took part in the research project. Forty-five final codes, generated from 89 codes initially obtained from the dataset, are classified into five final themes: uncertainty, overburdened, neglected, worry about students, and influence, which reflect the professional stress of primary school teachers following the epidemic prevention policies relaxed.

**Conclusion:**

Five themes were identified in the research. The problems described by the participants include burdensome offline activities, being disturbed out of hours, and appearing understaffed for the infection. These problems harmed the participants’ mental health, including anxiety, fatigue, stress, and other adverse psychological conditions. Awareness and attention to the psychological situation of primary school teachers after the eased COVID control are crucial. We believe protecting teachers’ mental health is necessary, especially in this particular period.

## 1. Introduction

At the end of 2019, COVID-19 spreads rapidly worldwide (Cao et al., 2020). Until now, the epidemic has not stopped. The three-year-long epidemic has harmed people’s physical health and caused psychological stress (Xiao, 2020). Many previous studies show the epidemic’s impact on people’s mental health. For example, in the study of Wang et al. more than half of the Chinese respondents said they suffered from moderate to severe psychological effects. At the same time, one-third had varying degrees of psychological anxiety symptoms Wang et al. (2020). Similar investigation reports have also appeared in the United Kingdom, Spain, Italy, India, and other countries (e.g., Dawson and Golijani-Moghaddam, 2020; Grover et al., 2020; Mazza et al., 2020; Rodríguez-Rey et al., 2020).

The COVID pandemic also affected school teaching. During the worst period of the epidemic, most countries have temporarily closed schools, so teachers have to start online teaching (Pokhrel and Chhetri, 2021). Changes in teaching methods, the teaching environment, other aspects, and some new job requirements have put psychological pressure on the teacher group, with some teachers experiencing psychological symptoms of anxiety (Pressley, 2021). Although teaching was considered stressful long before the COVID pandemic (Kyriacou, 2001). During this particular period, the need to complete many tasks online to ensure that teaching is carried out without interruptions and the need to take extra care of themselves and family members have undoubtedly added to the burden of teachers. People under prolonged stress are more likely to suffer from burnout (Embriaco et al., 2007). A study by Košir et al. (2015) revealed a positive correlation between teacher stress and burnout. Burnout not only negatively affects teachers’ teaching effectiveness (Schonfeld et al., 2019), but it can also harm physical health and increase the possibility of teachers suffering from depression (Martínez-Monteagudo et al., 2019). We should be more concerned about stress and burnout in the teacher group during the epidemic. However, relevant studies still need to be comprehensive. Some scholars even consider teachers to be forgotten frontline workers in the COVID pandemic (Beames et al., 2021).

Many countries have already adjusted their epidemic prevention policies (for example, China optimized its virus response and came up with a significant shift in its epidemic policy by downgrading COVID management at the end of 2022). However, there is a gap in the research on stress and job burnout among primary school teachers after the loosen res. Therefore, using various data collection methods, this study aims to investigate primary and secondary school teachers in China during this particular period. In detail, we hope to gain insight into the occupational stress of primary school teachers to explore the underlying factors that contribute to their stress. To seek answers to this question, 18 primary school teachers from different schools, genders, and lengths of service as teachers in Zhejiang Province, China, who experienced the epidemic, were selected for this study. With various rich data collected through various means, we obtained the teaching diaries of several teachers, which recorded their daily work and inner feelings before and after the epidemic. It ensures the authenticity of the data and can be used for in-depth qualitative analysis of teacher stress and job burnout. This study explores the factors influencing the occupational stress of primary school teachers in China after the shift in epidemic policy and contributes to expanding the research on teacher burnout.

## 2. Literature review

### 2.1. Teacher job stress and burnout

Kyriacou and Sutcliffe first proposed the concept of teacher work stress and defined it as the negative emotional experience of teachers in teaching work, such as frustration, anger, tension, etc., Kyriacou and and Sutcliffe (1978). Since the concept of teacher stress was introduced, scholars have researched this topic, and there have been a variety of methods to measure teacher stress. For example, from a physiological perspective, teachers’ stress levels can be determined by measuring their cortisol levels and resting heart rates (Roeser et al., 2013). Alternatively, assess teachers’ stress according to their uncomfortable experiences in the work environment (Schwarzer and Hallum, 2008); Another scholar developed the teacher stress scale and verified its reliability and validity (Fimian and Fastenau, 1990). Most researchers use questionnaires to investigate teachers’ stress (Kyriacou, 2001). For example, Desouky and Allam used a questionnaire to investigate the stress of more than 600 teachers in India’s primary schools and special schools. The results showed that all teachers who participated in the survey had varying degrees of work stress Desouky and Allam (2017). Some scholars have developed models of teacher stress. For example, Montgomery and Rupp (2005) proposed a theoretical and empirical model of teacher stress based on a literature overview and research analysis of stress. This model emphasizes the interaction between teachers’ internal experience and external pressure and takes an individual’s internal response to external pressure as the model’s core. They believe that individuals’ external pressure and internal experience form a “cycle” that will worsen if no appropriate intervention, which will affect teachers’ happiness.

There are many reasons for teachers’ work stress. We can find the source of stress in teachers’ daily working environment and the interaction between teachers and the people around them. First of all, the interaction between teachers and students and students’ performance has always been one of the causes of teachers’ stress. Antoniou et al. Investigated the working stress of 439 primary and secondary school teachers in Greece and found that problems in interaction with students and dealing with students’ bad behaviors were teachers’ main sources of stress Antoniou et al. (2006). Secondly, workload, working conditions, and requirements influence teachers’ working pressure (Roeser et al., 2013). In the United States, the federal government once issued some policies, including advocating for teachers to adopt teaching practices that meet standards, changing teacher evaluation methods (linking teacher performance with student test results), and so on. These policies have increased teacher demands, resulting in increased pressure on teachers (Von Der Embse et al., 2016). In addition, if teachers have problems getting along with parents, cooperative conflicts with colleagues, or lack of support from school leaders, the above conditions may aggravate their stress symptoms (Skaalvik and Skaalvik, 2007).

There are many negative consequences caused by stress, not only for teachers but also for students, schools, etc. First of all, from the personal level of teachers, excessive pressure is likely to threaten their physical and mental health and impair their performance in work and daily life (Asa and Lasebikan, 2016). A study conducted in several middle schools in Tanzania found that teachers’ stress level was related to violence against students. Excessive work pressure would aggravate teachers’ violent discipline against students (Hecker et al., 2018). In addition, work pressure is also one of the reasons for the loss of teachers (Gallant and Riley, 2014; McCarthy et al., 2014). For example, in a survey conducted in Jilin Province, China, nearly half of the Chinese teachers said they Would leave if future opportunities arose. Excessive work pressure was the main reason driving them to transfer jobs (Liu and Onwuegbuzie, 2012). Secondly, from the perspective of students, although no studies have pointed out that teacher pressure has a direct relationship with students’ performance or behavior, many studies have focused on teacher pressure and students. Some studies have shown that overstressed teachers often show a loss of motivation and passion for teaching and are less effective in teaching, thus reducing students’ interest and motivation in learning (Pakarinen et al., 2010). Furthermore, stressed teachers are likely unable to strengthen the relationship between teachers and students and provide necessary support or care for students on time (Hoglund et al., 2015). Finally, from the school’s perspective, excessive pressure has always been the main reason for teacher turnover. The loss of teachers is likely to make the school face the dilemma of a shortage of teachers, thus dragging down the overall teaching quality of the school.

Therefore, teachers must have good pressure regulation abilities. If teachers lack an adjustment pressure strategy or ability, long-term work pressure cannot be relieved, and it is easy to produce job burnout. Burnout was first proposed by Freudenberger (1974) to describe the emotional exhaustion he observed while working as a healthcare provider. Subsequently, some scholars defined burnout as a complete manifestation of emotional exhaustion, depersonalization, and low personal accomplishment (Maslach et al., 1996, p. 4). Maslach et al. (2001) identified emotional exhaustion as a major contributor to burnout—this kind of emotional consumption easy to makes individuals feel both physical and psychological fatigue at work. The characteristic of depersonalization is that teachers lose care for the object of work (such as students and colleagues) and show coldness and distance in getting along with the object of work. Low personal achievement will make teachers feel powerless and fail in the work process, reducing their work efficiency. Research on teacher burnout has been conducted in the United Kingdom (e.g., Hastings and Bham, 2003), the United States (e.g., Lee, 2017), Canada (e.g., Fernet et al., 2012), Italy (e.g., Carried out by Pedditzi et al., 2021) and other countries. Obviously, teachers’ job burnout is a concern of scholars in many countries.

### 2.2. The present study

Teaching is considered a psychologically demanding profession (Laybourn et al., 2019). Influenced by various factors, teacher populations often report high levels of stress and anxiety (e.g., Rubilar and Oros, 2021).

Pandemics provide a new context for teacher stress research, and understanding teacher stress in the context of a pandemic is imperative (Holmes et al., 2020). Although many scholars have explored teacher stress during the pandemic lockdown using quantitative, qualitative, or mixed methods (eg., Alqassim et al., 2022). However, few scholars have focused on teacher stress after the epidemic is released. And while most past research on teacher stress appears to have relied on quantitative approaches (e.g., using highly structured rating scales that include fixed response options), few approaches allow teachers to articulate their feelings or experiences of stress from their perspectives. However, such approaches often help researchers gain insight into teachers’ experiences of stress (Shernoff et al., 2011). Therefore, this study chose a qualitative approach to explore the occupational stress profile of Chinese elementary school teachers and their related experiences during the initial phase after the epidemic was liberalized. Our study aimed to investigate the following questions: How about the work pressure of primary school teachers during the initial phase of COVID restriction relief? What impact did the reopening have on the work of elementary school teachers?

## 3. Materials and methods

### 3.1. Participants

For this study, two recruitment strategies were employed. After explaining the study and our need for teacher participants *via* email, we sought the assistance of the principals of several elementary schools in Zhejiang Province to find teachers to volunteer for the study. Additionally, our research team shared information about the study and its participant recruitment strategies on social media (e.g., online teacher forums). The study participants were chosen based on the following criteria: (1)A regular faculty member who teaches in primary schools, (2) at least 3 years of experience; and (3) informed consent and voluntary participation. A total of 18 primary school teachers (9 male and 9 female) volunteered to participate in our study. The 18 participants were contacted by email to confirm that their personal information would be kept strictly confidential and that they could leave it at any stage.

For ethical reasons, the names of 18 elementary school teachers were anonymously transcribed into numbers. Table 1 shows the demographic characteristics of the participants in this study.

**Table 1:** Demographic features of participating teachers.

Teacher number	Gender	Experience
1	Male	5
2	Male	11
3	Male	13
4	Male	8
5	Male	10
6	Male	6
7	Male	20
8	Male	13
9	Male	15
10	Female	9
11	Female	7
12	Female	4
13	Female	10
14	Female	14
15	Female	12
16	Female	6
17	Female	3
18	Female	8

### 3.2. Data collection

We collected data from semi-structured interviews and teacher diaries during this research stage in January 2023, with the participants’ and principals’ permission.

The first source of data was semi-structured interviews with 18 participants (Seidman, 2005). Our researchers contacted them by email to set up an interview scheduled at a convenient time for the participants. Interviews were conducted from Monday, January 4, 2023, to Friday, January 10, 2023. In other words, interviews were conducted 4–5 weeks after the downgrade covid management. A combination of online and offline delivery methods was used for the interviews. Face-to-face interviews with participants from Hangzhou, Zhejiang Province, took place in the conference room of the Jing Hengyi College of Education at Hangzhou Normal University. There was a promise of silence and an absence of outside noise in the meeting space. We conducted online video interviews using Zoom for participants in other regions of Zhejiang Province. The interviews with each participant lasted 45 to 60 min and were taped with their permission. The interviewees’ real names were withheld to maintain their anonymity. The following is an outline of our interviews.

1. What is your inner feeling after the downgrade covid management?
2. Has your work changed since the downgrade covid management? If so, in what ways?
3. Do you think you have a heavy workload after the downgrade covid management?
4. After the downgrade covid management, have you encountered any complex work problems or troubles?
5. How do you feel about yourself after the downgrade covid management? How stressed out do you feel at work?
6. How has this eased covid control affected you (personally, emotionally, or otherwise) so far?
7. Do you have any other opinions regarding the pressure on the teaching profession now that the epidemic lockdown has ended?

Another source of data was the (teaching) diaries recorded by teachers in their everyday work. We asked the 18 participating teachers if they would be willing to share their most recent teaching logs, but not all agreed. We mainly focused on looking through the teaching diaries for records of stressful events, which allowed us to learn more about teachers’ stress burnout following the epidemic.

### 3.3. Data analysis

Qualitative data can be analyzed in several ways, and in this study, we used Braun and Clarke’s thematic analysis method to process the collected data (Braun and Clarke, 2013). The thematic analysis method includes the following steps: familiarizing with the data, generating initial codes, searching for themes, reviewing themes, defining and naming themes, and writing reports (Braun and Clarke, 2006). All interviews in this study were transcribed within 48 h of the interview. The researchers then invited participants to check to ensure that the transcriptions were consistent with their true thoughts, as suggested by (Creswell, 2008). The researchers imported all data transcriptions into a word document for generalization.

On the one hand, the Chinese relaxed Covid-19 restrictions led to an entirely new situation; on the other hand, the interview responses of the 18 participants varied in length, and the teaching diary data varied across participants. Based on these two reasons, we assume it is appropriate to adopt this method. We used data and methodological triangulation to ensure the validity and dependability of the study results. To be more precise, our researchers chose participants for this study from different genders and teaching ages. We used various methods to collect data (including interviews and diaries), ensuring the data’s richness and diversity. Our researchers read the interview transcripts and teaching journals numerous times as they immersed themselves in the data, made their initial observations of potential themes, and finished the initial coding of the data. Checks were organized after codes were created as a way to prevent code duplication and prevent data bias. Our research team uses telephone communication to resolve conflicts when they arise. The final topic is chosen after several iterative drafting and redrafting sessions.

## 4. Results

Our thematic analysis identified five themes: (1) uncertainty, (2) overburdened, (3) neglected, (4) worry about students (5) influence. These topics collectively describe the experiences of 18 teachers 3–4 weeks after the Chinese government relaxed the Covid-19 restrictions.

### 4.1. Theme 1: uncertainty

**Figure 1:**
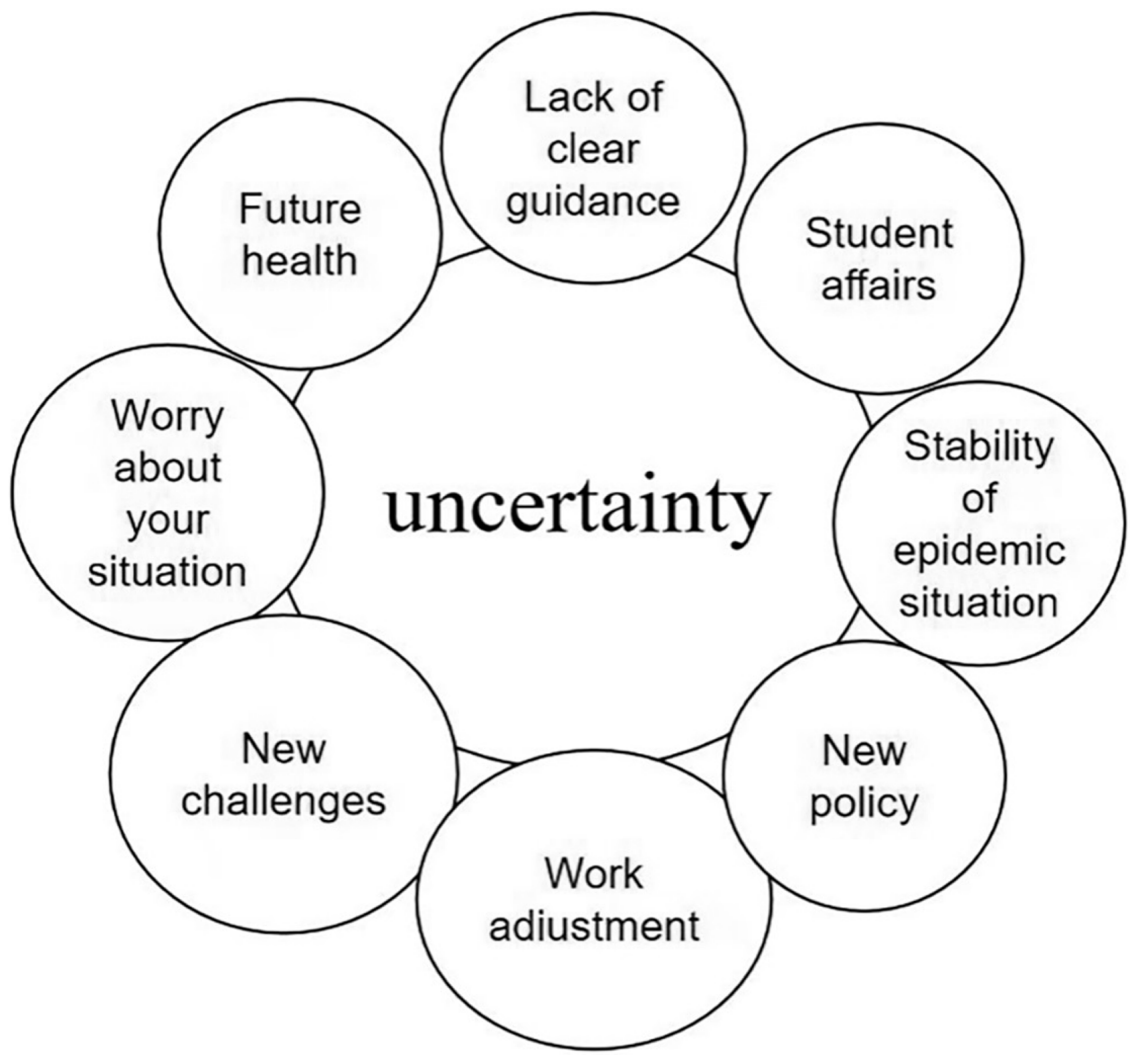
The theme uncertainty and the codes it consists of.

**Figure 2:**
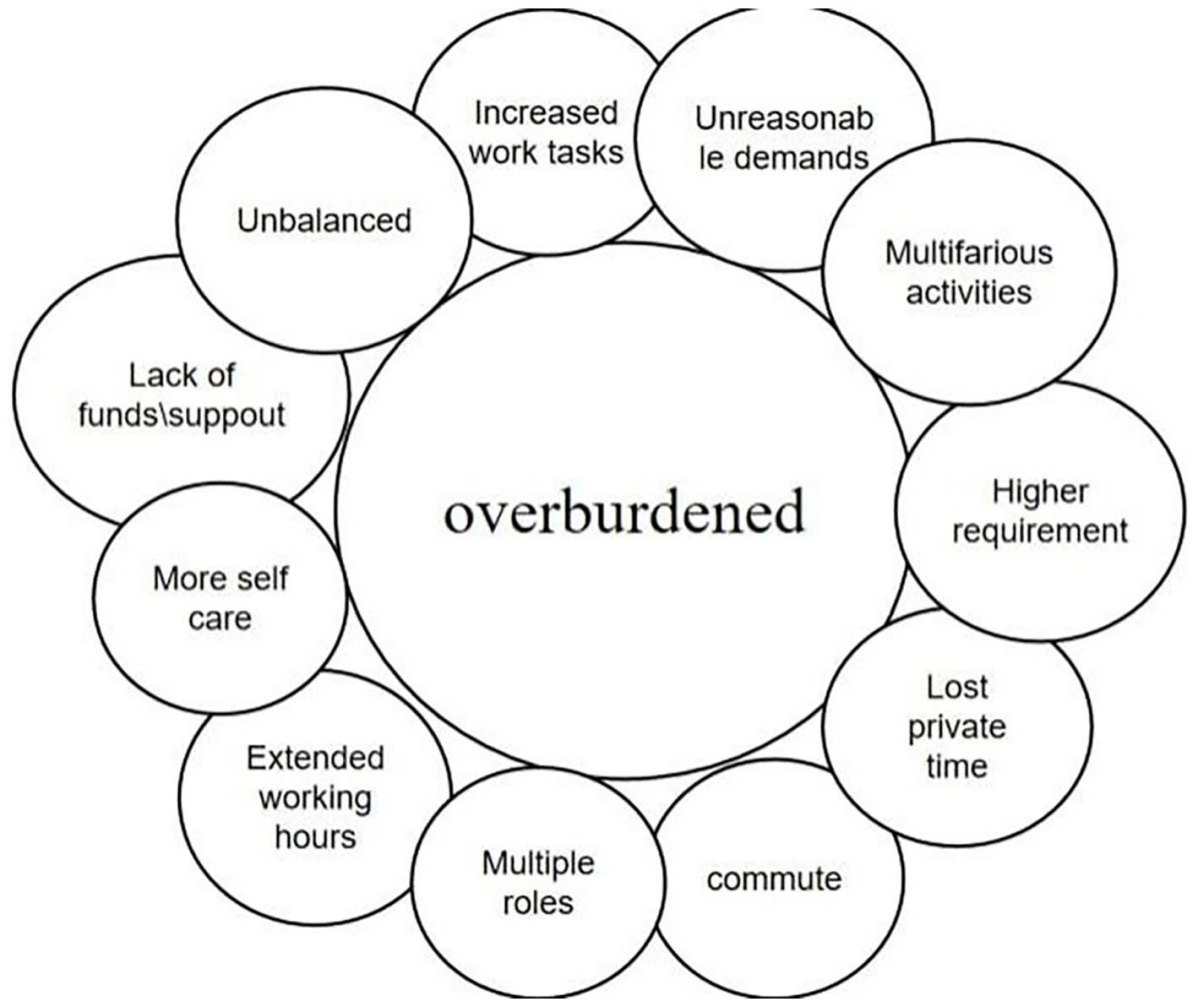
The theme overburdened and the codes it consists of.

**Figure 3:**
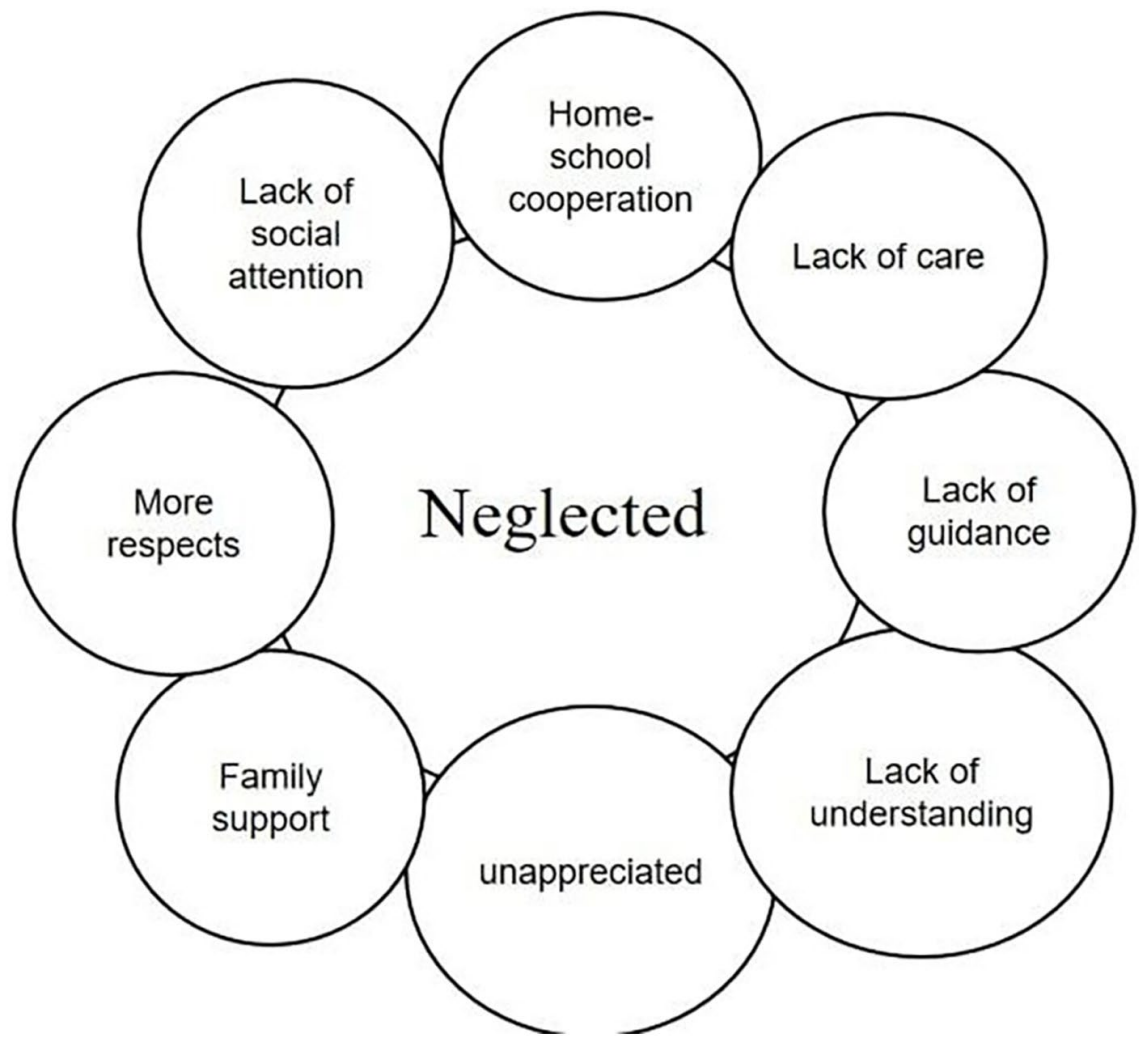
The theme neglected and the codes it consists of.

**Figure 4:**
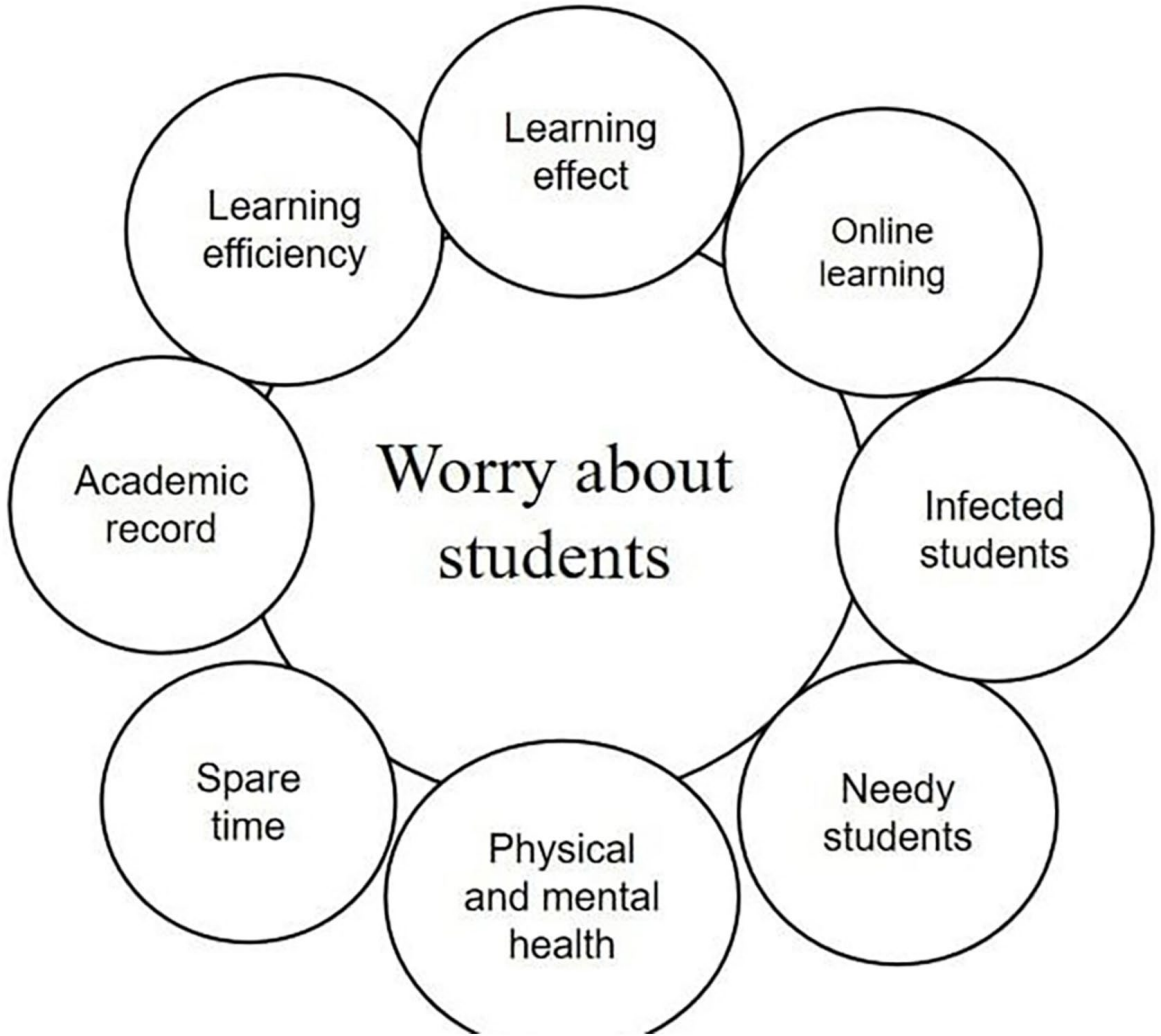
The theme worry about students and the codes it consists of.

**Figure 5:**
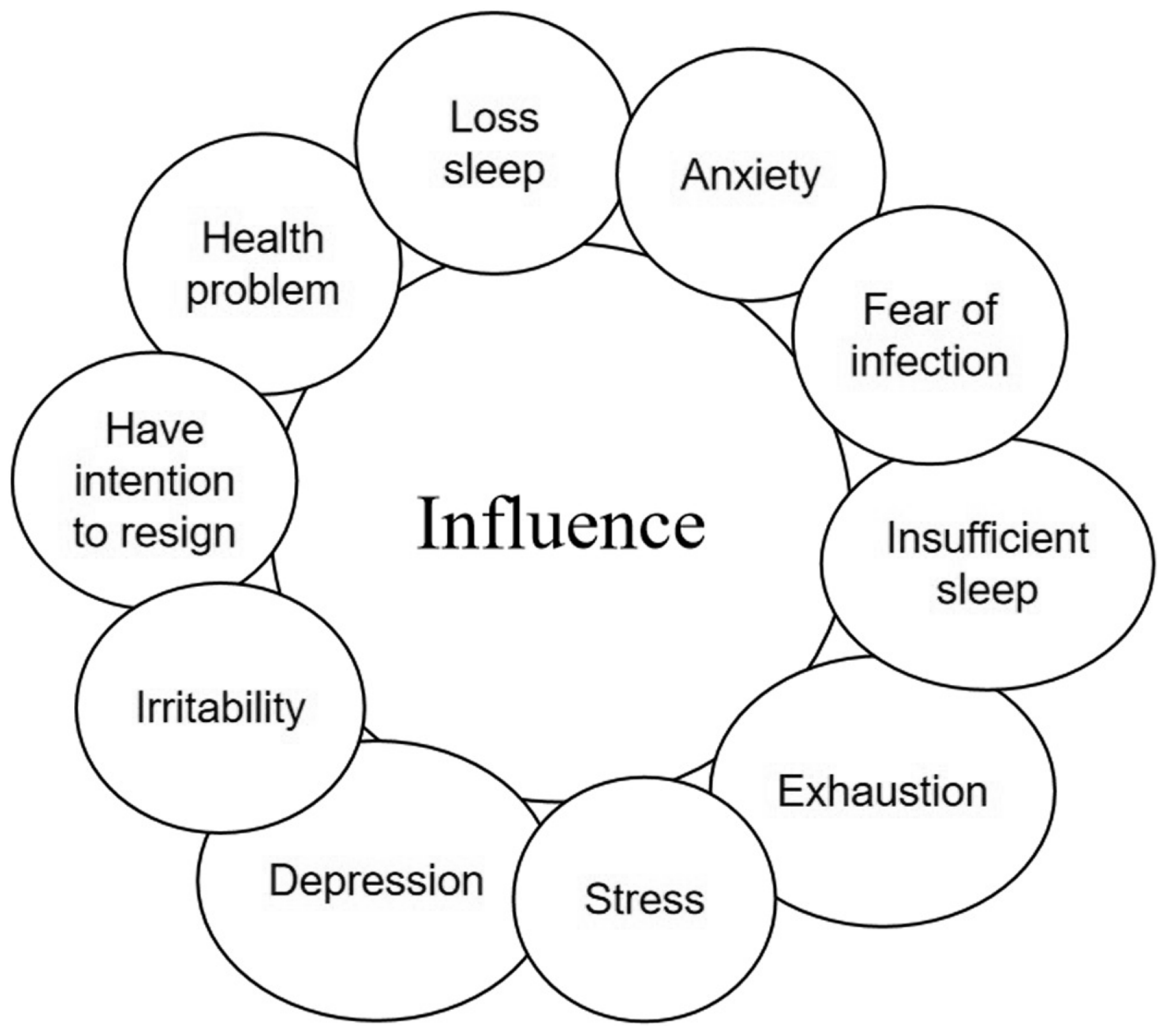
The theme influence and the codes it consists of.

This theme reflects teachers’ uncertainty about job adjustments in the first days after the government declared the epidemic restriction eased. Participant 11 indicated that this feeling was difficult to articulate. Imagine you have spent time and energy adjusting to your work under regular epidemic prevention and control measures. Suddenly, you must make changes and return to your old teaching job. However, you are unsure if you are completely back to your old self (see Figures 1–5).

In most cases, the initial reaction is similar to being at a loss. Downgrade COVID management will mean a specific shift in your daily routine and habits of the past 2 years and an adjustment in your work status, tasks, etc. It is like taking you from one familiar place to another, and it can feel like being caught off guard. This analogy of going to an unfamiliar place was also mentioned by two other participants, 4 and 18. Participant 1 detailed the multiple factors that led to his uncertain feelings in her teaching journal during this time.

“Since lift restrictions, most people are cheering. But my colleagues and I feel nervous that in casual conversation, we are unsure what adjustments will be made to school teaching after the policy change. Would we be faced with a new workload? Should we still use distance learning for instruction? Will there be policies in place to make adjustments at a later date? Furthermore, downgrading the COVID management makes me even more worried about the health situation. Because many students interact face-to-face in the classroom, the space is a little small. Once there is one infected child in the class, there is a high risk that this child will also infect other uninfected students with the virus.”

However, some teachers consider this uncertainty as a new challenge. They looked forward to the challenge of uncertainty and felt they could easily handle the new adjustment. This group of teachers was primarily those who had started earlier and had more experience. For example, Participant 9 said, “This is a new situation, and I think my work in the 3 years under epidemic control will be adjusted and changed to a great extent. Lift restrictions signal to us educators that we need to tune in to ourselves as soon as possible and be ready to listen to the arrangement of the higher management. Despite the uncertainty, I am excited about this.” In this context, one teacher described the sense of accomplishment in meeting the challenge of opening up to the epidemic: “I’ve had several discussions with the principal and chairman in Academic Affairs Office this past week, and the main discussion was what we need to do with this epidemic opening up. Not only just the teacher but also the home-school cooperation and other aspects we have considered, and I think we have identified most of the things that we need to do, and we can even say that we are well prepared.” (Participant 7).

An aspect of uncertainty was a self-consciousness about one’s circumstances. After the epidemic prevention policies change, teachers must work with their managers to adjust their workload appropriately while also considering their health. I’m concerned that I will be in a hurry and unsure whether I will be infected during the first stage of the opening up (Participant 5). It became too much for teachers to protect their students while looking after themselves and their families.

“After the loosen COVID curbs, I did not receive detailed work arrangement or notice from the school. Several parents have called me to ask about their concerns, such as lunch breaks, group meals, etc. I know that parents want to know about the school’s efforts, that is, how much effort the school will put into their child’s safe schooling, as it is obvious that no parent would like to expose their child to the risk of infection. But I cannot give students’ parents a clear answer, making me nervous and uncomfortable. And if my family or I develop an infection later, I will need to put in the time and effort to take care of it. If I had an infection and could not teach the students, I would be guilty of the delay in teaching because of me.” (Participant 10, from teaching diary).

The theme of uncertainty also reflects teachers’ need for more clarity in policy. More than half of the teachers would like advice or targeted policy guidance from their local education department to help them plan and organize.

According to participant 12, “We all need clear rules or guidelines from our supervisors right now since not only the teachers but also the pupils and parents are uneasy with this ambiguity. For instance, teachers should be allowed to participate in decision-making processes and always be able to communicate with their managers about issues they are having. To avoid negative teaching issues after the opening up, primary and secondary schools in the same area should work together to communicate and support one another.”

### 4.2. Theme 2: overburdened

It is worth mentioning that the researcher’s initial thought was that the burden of teachers would be reduced after the epidemic prevention policies relaxed, yet the actual survey was the opposite. The 18 participating teachers said they felt overburdened. Some participants described to us sources of burden, including increased work tasks and longer working hours. Of course, some sources of stress mentioned above, such as lack of financial support, had existed before the epidemic. But the changes in work hours and tasks were related to this shift in the epidemic policy.

Some participants mentioned that less than a week after the shift in the epidemic policy, colleagues in the office were infected. The shortage of staff seemed to be more severe than before. “At first, the infected teachers did not know they were infected with COVID-19 until they had fever symptoms and went to the hospital for nucleic acid tests. Then several other teachers in the office had to be isolated at home due to frequent contact with the infected person.” “More than 10 teachers in the school have been infected, and I feel a bit heavy when I see colleagues coming home for isolation one after another. On the one hand, I am worried about the health of my colleagues. On the other hand, the teachers remaining in school is decreasing, leading to staff shortage in school work; this also makes the workload of our teachers who have not been infected more than before.” (Participant 8, excerpted from the teaching diary) Participant 5 expressed that the downgrade covid management increased the possibility of infection, which made him panic. “Because there are no health code checks, mandatory masks, or nucleic acid anymore, we are likely to be infected on the daily commute.”

Other participants gave a different reason. “Before the epidemic was liberalized, most lessons were in the form online. I think online teaching is relatively easy for young teachers like me. The lesson time was reduced from 40 to 20 min, and there was no need to manage class discipline, safety, and hygiene issues. After the epidemic was liberalized and changed to offline teaching, classroom management became more difficult. Especially managing the younger students requires personal attention to everything, and they must be stationed in the classroom between classes. On average, there are four classes every day. And there are six classes daily if you add the morning reading and lunch control. When I get home, I must prepare lessons and copy notes. So, I am exhausted and have little time for myself” (Participant 17). “When I worked at home, I could teach online just after waking up and turning on the computer. But now I have to get up an hour early and take the subway to school. Especially when it is my turn to be the duty teacher, I have to get up earlier and earlier and leave later and later.” (Participant 18).

This was especially true regarding the extra workload that came with the reopening of the epidemic policy (e.g., leave processing and disinfection in classrooms). One participant said, “During the school’s closure, we uniformly conducted distance education at home, but now we must consider both online and offline teaching. Due to the liberalization of the epidemic, some students are infected and studying at home. In contrast, those who are not infected continue to come to school, so we need to take two measures according to the situation of these two types of students. The learning progress of the two should be consistent, and the homework should be corrected separately (students at home should upload it in the form of photos, and students at school should submit it directly)” (Participant 2). When describing how she felt when she had more job demands, one participant wrote in her diary, “Tasks like sanitizing the classroom should be done by specific people instead of leaving it all to the teacher, who should be allowed to focus on the lesson.” (Participant 6). Another participant described various school offline activities as one problem, “I do not need to fill out forms every day. However, some offline activities could not be carried out due to the pandemic lockdown. Now that the epidemic lifted, many offline activities such as parents’ Open Day, public classes and classes where novice teachers appear have been arranged in a short time.” (Participant 1, from teaching diary).

Some other participants described being frequently interrupted during non-working hours as a contributing factor to overload. Schools are often given new tasks at the beginning of the liberalization of the epidemic. Many parents of students, who often ask teachers for help, worry about their children’s health and their performance at school. One participant said, “After the epidemic was liberalized, I often received phone calls or messages from the principal, colleagues, and parents on weekends. And I often worked late into the night because of one of them. Although this situation also occurred before, the reopening of the epidemic policy has not changed it. This has put a damper on my work-life balance.” (Participant 4). “I am tired from working all day but cannot refuse to answer the phone. I have to respond to the phone of the principal and parents with enthusiasm.” (Participant 11, from teaching diary).

It is worth mentioning that even though teachers reported feeling overburdened after the epidemic was liberalized, a small number of teachers said they also had some positive feelings. For example, compared to the period when the school was temporarily closed, teachers at least had more face-to-face interaction with their colleagues and the principal. These factors reduce their burnout. One participant said, “I have been working with my colleagues since the reopening of the epidemic policy. We often help and encourage each other, so I am not alone. The school even organized a small campus reading activity a few days ago. I think all of them have helped me avoid burnout as much as possible” (Participant 7). “It is not compulsory to make nucleic acid!” (Participant 13) One said, “I feel like everything is slowly recovering after the epidemic was lifted” (Participant 12, from teaching diary).

### 4.3. Theme 3: neglected

Some teachers also mentioned the feeling of being neglected. The responses of the participating teachers showed that they felt they were neglected after the epidemic was liberalized, especially by the school leaders and the relevant education departments. Participants felt that school leaders and relevant education departments lacked attention to the teacher community after the epidemic was lifted. “The local education department should develop relevant policies regarding teacher support after the epidemic was lifted!” (Participant 9) Participant 3 wrote in her teaching diary, “Since downgrade COVID management, school leaders have focused more on the students who have been infected with the virus but have rarely asked whether our workload is too heavy and whether there are difficulties during the special time. Only the constant daily flow of task notifications from upper-level administrators made me feel like a machine.”

Participants also raised issues such as not all parents of students cooperated with teachers during the period after the loosened restriction and that some extra work teachers took on after the opening up was taken for granted and not appreciated and acknowledged accordingly. After the epidemic prevention policies were relaxed, teachers shifted from online to blended teaching. Both current and quarantined students were required to attend classes and correct assignments, and participants described the challenges and stress of coping with the new situation under inadequately supported conditions. “This situation made teaching difficult, and we needed to immediately switch from our past online teaching style, often staying up late to adjust lesson plans. However, some students’ parents did not cooperate well with teachers, making our work even more difficult. Not getting parents’ understanding and compassion made me lose motivation for teaching” (Participant 14, from teaching diary). “Instead of showing gratitude to us, parents worry about their children’s health or performance and take it out on teachers.” (Participant 5).

In addition, other participants reported that they had developed symptoms of infection soon after the downgrade in COVID management and that their condition had resolved within a week or so. However, due to the infection, the backlog of teaching duties increased their stress levels. “Although the condition has improved, the body is still recovering. The overload from the infection can be a serious health hazard, but the teaching work left me feeling out of breath. And I am afraid that I will get infected again.” (Participant 16)

### 4.4. Theme 4: worry about students

Most teachers have expressed concern for their students, especially those with an infected person in their home. This issue seems to cause more anxiety among teachers than any other. Teachers’ concerns about students fall into several categories. Some teachers worry about the physical and mental health of their students. Participant 6 mentioned that “students’ resistance is not as good as adults. Although they spend most of their time at school and home, their parents spend some time in public places due to their work. Several parents in my class have already been infected, and they could have accidentally passed it on to their children. I’m apprehensive about my students.” Other teachers described emotional instability among some students after the release of epidemic control, which appeared to be worse than during the lockdown. Students need more care and guidance from teachers than ever before. For example, participant 15 wrote in his teaching diary that “the release of epidemic control has had an impact on student’s mental health. I could feel their unease and fear in my daily conversations with students. Especially when a student was infected in the class, even the students in the class next door were in a panic, so I felt the need to strengthen education on this.” “Some students seem very angry and always speak angrily to the teacher.”

Some teachers worry about their students’ learning. Participant 18 believes that the release of the epidemic control has caused an inevitable panic among students, which is bound to affect their daily studies. “Especially for the infected students, the high fever for several days can make it difficult to follow the normal teaching schedule.” In addition, students’ activities were restricted before due to the epidemic, but now some students take it as a signal of “liberation” after the epidemic control was eased. Some teachers are worried that students will get into fights and enter adult Internet cafes when they return to school. “I understand that students have been depressed for too long and want to go out to play, but I also worry that some students will not work as hard as before after opening up, which means there will be a kind of revenge libertinism. The Internet cafes and game arcades outside the school are gradually opening up, so they will probably go there after school.” (Participant 7).

In addition to physical and mental health and learning, the teachers also pointed out that some students have poor conditions in online teaching due to their family conditions, namely, the unfavorable home learning environment. “Teaching after the opening up is not completely transformed into offline teaching. For example, if some students are infected, they need to go home and study through online courses. Students from low-income families in my class must piggyback on their neighbors’ Wi-Fi for online learning.” (Participant 8).

In short, worry about students was a common denominator for most participants.

### 4.5. Theme 5: influence

The participants’ feedback showed that the epidemic prevention policies change had some impact on their emotional and mental health. All participating teachers described different feelings, such as stress, anxiety, worry, and exhaustion. One participant described her stress and anxiety in her teaching diary “I have a school open class presentation coming up soon, and the head of the year group sent me as a representative. I have to manage the class during the day with various matters waiting for me, and I must stay up late at night to prepare and polish the lesson.”(Participant 12, from teaching diary). Another person described exhaustion and burnout: “Although the epidemic prevention and control have been relaxed, my work had not been relaxed. I also had to keep in touch with school leaders and students’ parents, and I lost time for myself. I had two cups of coffee daily but still felt exhausted.” (Participant 3). Other participants described the imbalance between work and family in their teaching diaries, “I was so busy during the time, some of the work adjustments were hard to adjust to. Like being on a see-saw, I could not balance work and family. My workload left me with very little time to spend with my family. Every day when I came home, I was silent, did not have the energy to do housework.” (Participant 11). Interestingly, the female participants mentioned the feeling of work-family imbalance several times. Participants also described the impact of stress from school, society, and parents of students on their mental health. Participants described being pressured by multiple sources when the epidemic prevention policies were relaxed. For example, one participant described pressure from parents, “Several parents have contacted me privately to ask for special attention for their children, such as having their children eat alone on school grounds to reduce contact with other students. This puts me in a dilemma, and I have been losing sleep lately.” (Participant 8, from teaching journal).

A few participants said they might leave their frontline teaching position to pursue other education-related jobs. They felt that they already felt stressed and exhausted during the pandemic, and the period of epidemic liberalization did not relieve them of these psychological conditions. “Often, I calculate with my colleagues around me how long it will be before I retire. Retirement would not be as much work to deal with.” (Participant 9). “I was already tired from the epidemic in the past 3 years, and during this time, I feel my enthusiasm is worn out, and I have some health problems, so I will consider changing my career.” (Participant 6).

In addition, participants expressed a desire for more teacher counseling and coaching services in the future.” There are general student counseling rooms in schools, but few teacher counseling rooms. I hope that in the future, schools will provide services for teachers in this area and care about the mental health of the teacher community, especially after the epidemic Easing Restrictions.” (Participant 7) “The psychological condition of teachers affects the quality of teaching work and should be taken seriously by both schools and relevant education departments.” (Participant 15) “I would feel much lighter if professional people could listen to our work stress and anxiety and channel them to us.” (Participant 17)

## 5. Discussion

To the best of our knowledge, this is the first study to investigate the occupational stress and influencing factors of primary school teachers in China after the epidemic was lifted. In the weeks after the announcement of the policy, we learned about the psychological pressure of primary school teachers in China related to the release of the epidemic through interviews, reading teaching diaries, and other ways. Using thematic analysis, we identified five topics related to occupational stress: uncertainty, overburdened, neglected, worry about students and influence.

Our findings suggest that some primary school teachers still have symptoms of stress in the early stages of opening up. This finding is consistent with previous international studies on the lockdown phase of the pandemic, which showed high levels of stress, fatigue, and cynicism among school teachers affected by the pandemic (Sokal et al., 2020; Rubilar and Oros, 2021). A quantitative study in Spain revealed that psychological symptoms such as anxiety and stress suffered by teachers are not only characteristics of the lockdown stage (Ozamiz-Etxebarria et al., 2021), which coincides with our study. In our survey, participants repeatedly described their sense of being overburdened in the stage of COVID-19 restrictions were fully lifted. For example, offline activities such as parents’ Open Day and public class demonstrations, which could not be held as scheduled due to the epidemic, have surged in the post-pandemic period. All kinds of school activities are scheduled to be packed. Due to the pandemic, the lack of staff in schools has led to uninfected teachers sharing more of the workload. This reminds us that even though most countries have relaxed epidemic control, teachers are still under the shadow of the epidemic.

However, we should pay close attention to the driving factors that caused teachers’ stress in two different periods: the opening up and the lockdown of the epidemic. Comparing the two periods, there are similarities and differences in the driving factors that lead to teachers’ stress. Teachers’ pressure mainly came from the following four aspects during the lockdown period. At the working level, the forced transition from face-to-face to distance teaching brings challenges and pressure to teachers due to school closures (Pace et al., 2022), which is a significant point mentioned in many studies. First of all, some teachers had not mastered the technology or method of distance teaching before the pandemic, and teachers in some remote areas rarely used remote teaching equipment in teaching. Therefore, being required to be proficient with various online teaching devices and software in a short time has a negative impact on teachers, most of whom feel that they are not prepared to adapt to this new requirement (Goldschmidt, 2020). Secondly, there are many difficulties in the distance learning process, and teachers’ workload is more than before. For example, the school’s online learning system is not perfect. Some students cannot use the computer, and teachers must deal with network instability or interruption in the teaching process and other situations. In particular, teachers need to make more efforts to communicate with students who fail to connect to the Internet (Klusmann U. et al., 2022). In addition, the anxiety about teaching quality has increased the psychological burden on teachers. In a qualitative study to understand teachers’ anxiety about live teaching, some participants said due to the lack of face-to-face interaction, teachers are unsure if students are paying attention in class (or dozing off behind a screen). During online discussions, some students tend to copy others’ answers, which makes teachers uncertain about teaching effectiveness, thus generating anxiety (Liu et al., 2022).

From the perspective of personal life, teachers spend much time on online teaching, and some parents will disturb teachers during non-working hours out of concern for their children, thus blurring the boundary between professional work and the private life of teachers (Klusmann B. et al., 2022). For some female teachers, engaging in family activities (including caring for children at home or caring for infected families) and working from home bring about role conflict and increase their stress (Clark et al., 2021). Regarding physical health, teachers are ordinary people at risk of infection. Some studies have shown that teachers suffer from sleep deprivation and poor health during the pandemic (Souto-Manning and Melvin, 2021). At the height of COVID-19, teachers’ worries about physical health and threats to their health are potential stressors. From an emotional perspective, early studies have pointed out that using ICT at home will make people feel nervous, anxious, and tired (Cuervo Carabel et al., 2018). Teachers also have to fight the loneliness of being isolated at home. Moreover, during the lockdown period, the distance between teachers and students was far away, making it difficult for them to communicate emotionally. All of the harm mentioned above affects teachers’ emotional development.

Now China has downgraded COVID management, schools gradually return to offline teaching, and teachers have less time for distance teaching than before. Participants in the study cited stress drivers from the Initial stage of opening up:

1. The change of teaching plan and the shortage of staff. As the number of infected people (including teachers and students) increases after the eased COVID restriction, teachers must teach online and offline.
2. Uninfected teachers in the school should share the workload of infected teachers.
3. Unlike the lockdown phase of the epidemics, many offline activities were arranged in the short term after the opening of schools, making it difficult for some teachers to adapt.

Concerns about psychological problems or lousy behavior among students have also emerged as a new source of stress for teachers since the COVID restrictions were lifted.

Interestingly, some of the young participants in this study mentioned that they had a positive attitude toward the previous phase of distance teaching. Learning or using various online teaching devices and software was not particularly difficult for them. Participants mentioned the advantages of distance teaching that were less discussed in previous studies. On the one hand, teachers only need to focus on completing teaching tasks in distance learning and do not need to spend time and energy managing students’ lunch breaks, extracurricular activities, or other work. On the other hand, distance learning greatly reduces the pressure of teachers’ commuting.

Teacher well-being is critical to school improvement and education reform (Parker et al., 2012). Our findings suggest that primary school teachers in China are still under pressure in the initial phase of the COVID restrictions eased. In the post-COVID-19 era, it is necessary to take corresponding measures to relieve the pressure on teachers, improve their well-being and promote the development of the teaching profession.

## 6. Limitation

There are some limitations to the current study. First, the study’s sample size is small, and the lack of randomization limits the generalization of results. In future studies, the sample size should be expanded to obtain more universally relevant results, such as drawing representative random samples from different regions of China. Second, because participation is voluntary, it is not excluded that the participants in this study are teachers who are particularly affected by the opening up. Overall, however, the study used qualitative methods to understand the stress of primary school teachers in China during the initial phase of the pandemic and captured their expectations that schools would provide relevant mental health services in the future.

## 7. Conclusion and future research

To date, there have been no studies in China analyzing the occupational stress of elementary school teachers after the epidemic was liberalized and students returned to school, which is the main strength of this study. Our study examined the occupational stress of Chinese elementary school teachers in the initial phase after epidemic liberalization using interviews, a review of teachers’ diaries, and thematic analysis, which has practical implications for subsequent efforts to reduce occupational stress and burnout among elementary school teachers. Participants in this study described the negative effects they experienced at work during the initial phase of the epidemic’s liberalization, highlighting the experiences of overload and neglect—high levels of stress, exhaustion, and anxiety. Meanwhile, a small number of teachers have expressed a desire to resign. The many stressors will likely impact the professional well-being of elementary school teachers in China. The epidemic’s impact may be more significant than we can imagine, and the lasting damage reminds us that we cannot ignore the well-being needs of the teacher community. Therefore, in the post-epidemic era, there is a need to continue to study teachers’ stress and its influencing factors in various ways, which will help us take more targeted measures to alleviate teacher stress and burnout.

In the post-epidemic period, or what we might call the “restoration” period, it is necessary and beneficial for schools and administrative departments of education to focus on the daily work and physical and mental health of the teacher community and to provide them with some support. This support can include the followings: First, in terms of work, schools need to reduce the burden on teachers and implement flexible work arrangements (e.g., flexible schedules and shift work) as appropriate (Moreira et al., 2019). The second aspect is psychological. The epidemic has caused severe psychological trauma, and teachers are no exception. The 1,479 participants in the study by Alves et al. (2020) teachers experienced decreased emotional well-being (more stress, tension, anxiety and distress) compared to the pre-pandemic period. Participants in our study mentioned that they want schools to provide them with psychological counseling services. Therefore, schools can provide counseling services for teachers by opening teacher counseling rooms, stress relief rooms, online mental health platforms, and 24-h hotlines to assist teachers in releasing work stress and positively coping with psychological problems. This initiative would help improve teachers’ psychological resilience.

In our present study, some of the young teachers indicated that they did not reject distance learning and felt they showed high resilience to it. Technology stress is an under-explored area (Li and Wang, 2021), and future research could focus on technology stress. For example, teachers’ age, teachers’ mental health, and technology pressure can be combined to explore the relationship between the three aspects, and it will be interesting to deeply understand the attitude of teachers of different ages and different grade levels towards the integration of technology into teaching and their perceived technology pressure. Also, thinking about how to improve teachers’ ability to use digital teaching devices is a direction worth exploring in the future. The pandemic will be a thing of the past, but its impact on education is far-reaching. The pandemic has uncovered a shortcoming in traditional teacher training: There is still much room for improving teachers’ digital literacy. Many teachers have underperformed during the pandemic due to the inadequate support they have received for information-based teaching and learning. Teachers also need to be nurtured. In the future, it would be beneficial to consider incorporating programs related to information-based teaching into teacher professional development programs and pre-service teacher training to improve the digital literacy of the teaching force.

### 7.1. Moral statement

Studies involving human subjects were reviewed and approved by the Ethics Committee of Ethics Committee of the Jing Hengyi School of Education, Hangzhou Normal University, with an Ethics approval ID of 2,023,010. Patients/participants provided written informed consent to participate in this study.

## Data availability statement

The original contributions presented in the study are included in the article/supplementary material, further inquiries can be directed to the corresponding author.

## Ethics statement

The studies involving human participants were reviewed and approved by Ethics Committee of the Jing Hengyi School of Education, Hangzhou Normal University. The patients/participants provided their written informed consent to participate in this study. Written informed consent was obtained from the individual(s) for the publication of any potentially identifiable images or data included in this article.

## Author contributions

YY and JX: conceptualization. YY: data collection and analysis, draft writing. JX: contributed to the conceptual framework and supervision. All authors contributed to the article and approved the submitted version.

## Conflict of interest

The authors declare that the research was conducted in the absence of any commercial or financial relationships that could be construed as a potential conflict of interest.

## Publisher’s note

All claims expressed in this article are solely those of the authors and do not necessarily represent those of their affiliated organizations, or those of the publisher, the editors and the reviewers. Any product that may be evaluated in this article, or claim that may be made by its manufacturer, is not guaranteed or endorsed by the publisher.
